# Vocal coselection in rat pup ultrasonic vocalizations

**DOI:** 10.1002/ece3.1907

**Published:** 2016-02-24

**Authors:** Heather R. Spence, Ali M. Aslam, Myron A. Hofer, Susan A. Brunelli, Harry N. Shair

**Affiliations:** ^1^ Biopyschology and Behavioral Neuroscience ProgramThe Graduate Center of the City University of New York365 5th AveNew YorkNew York10016; ^2^ Department of Computer ScienceBoston College140 Commonwealth AvenueChestnut HillMassachusetts; ^3^ Division of Developmental NeuroscienceNew York State Psychiatric Institute & Department of PsychiatryColumbia University College of Physicians and Surgeons630 W 168th StNew YorkNew York

**Keywords:** Behavior, development, heritability, selective breeding, signaling, ultrasonic vocalization

## Abstract

Selective breeding and natural selection that select for one trait often bring along other correlated traits via coselection. Selective breeding for an infantile trait, high or low call rates of isolation‐induced ultrasonic vocalization of rat pups, also alters functions of some brain systems and emotional behaviors throughout life. We examined the effect of breeding for call rate on acoustic parameters that are of communicative significance. Selecting for higher call rate produced calls of significantly increased amplitude and bandwidth relative to a randomly bred line. Selecting for lower rate produced calls of decreased duration. These nonmorphological, functional trait changes demonstrate enhanced communicatory potential and energy expenditure for the High line and the opposite for the Low line. This demonstration of coselection in a communicatory system suggests an underlying heritable suite of linked acoustic vocalization characteristics that in noisy environments could enhance dam–pup communication and lead to selection of emotionality traits with beneficial responses to stress.

## Introduction

That selective breeding for one trait results in alterations of other traits was highlighted by Darwin as an important area of investigation, yet many aspects of coselection remain unclear in both laboratory and natural conditions (Hofer et al. 2001). How traits are related and the scope of the linked characteristics provide important clues about underlying mechanisms as well as evolutionary significance. For morphological traits, genetic integration is defined as a population‐level process, influenced by developmental integration (Cheverud 1996). Nonmorphological traits can also be inherited, yet much remains to be clarified about their functional, developmental and genetic integration. While some morphological traits like the coordination of hindlimb and forelimb formation have been described in detail (Cheverud 1996), nonmorphological trait studies have mainly focused on the relationships between selected traits such as anxiety or aggression with other behavioral or physiological characteristics (Castanon and Mormede 1994; Hogg et al. 2000; Nyberg et al. 2003). Traits like communicatory vocalization have been mentioned in a few such studies but without analyses of acoustic parameters (e.g., Formanek et al. 2011; Beausoleil et al. 2012). Here, we report on the effects of a long‐term breeding study for vocalization rate on other vocal characteristics.

Rat pups communicate with whistle‐like vocalizations in the ultrasonic range, between approximately 20 and 90 kHz (Roberts 1975; Branchi et al. 2001). These vocalizations change as pups develop toward adult rat acoustic communication (Bell 1974; Brudzynski et al. 1999; Brudzynski 2005). Studies of rat pup ultrasonic vocalizations (USV) in a variety of contexts, including isolation‐induced calling, contribute to research efforts investigating emotion, early‐life social interactions, attachment behavior, mother–infant interactions, juvenile behavior, and development (e.g., Amsel et al. 1977; Branchi et al. 2001; Hashimoto et al. 2004; Wohr and Schwarting 2007; Ise and Ohta 2009; Shair et al. 2009). Isolation‐induced USV has been considered a marker for anxiety as its rate is increased and decreased by anxiogenic and anxiolytic agents, respectively (Winslow and Insel 1991; Brunelli and Hofer 2001). Communicatory function has been demonstrated by observing adult response to infant calls (Smotherman et al. 1974; Brunelli et al. 1994; Rohitsingh et al. 2011).

**Figure 1:**
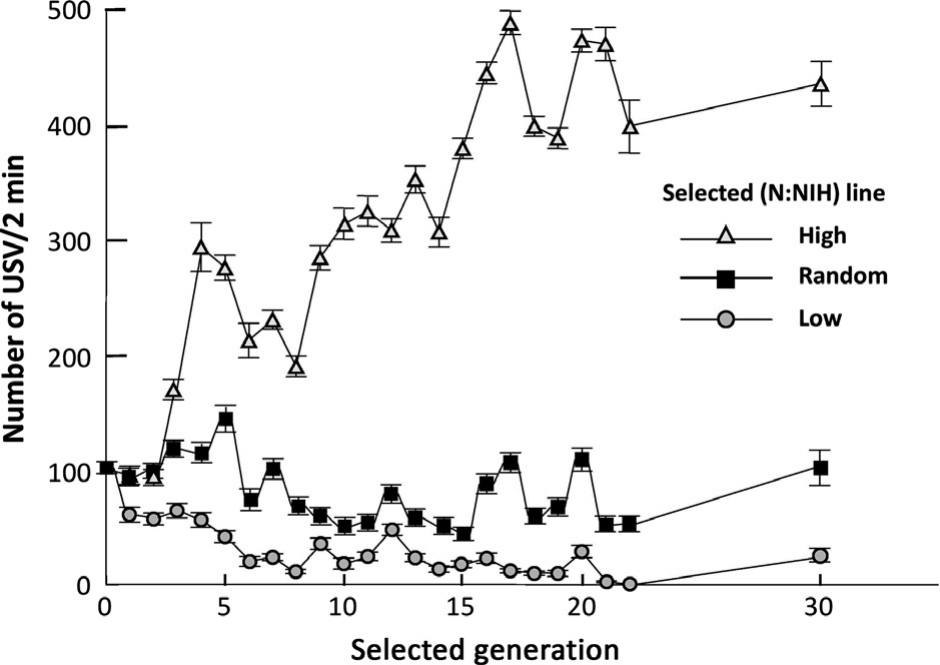
High, Low, and Random lines were selectively bred based on the number of USVs emitted during isolation from the dam, at room temperature. The Low line (circles) diverged from the Randomly bred control line (squares) in the S1 generation and the High line (triangles) diverged from the Randomly bred control line in the S3 generation. Data presented are for litter means. From Brunelli et al. (2010), reprinted with permission.

To provide insights into how biologic systems become recruited and integrated during ontogeny as expressions of temperamental affective responses, our laboratory carried out the first mammalian study to select extremes of an infantile trait. Two lines of rats were bred for higher or lower USV rate by 10‐day‐old pups isolated in a novel environment. Based on observations of these lines over generations, the rate of isolation‐induced USV is heritable (Hofer et al. 2001). The High and Low vocalizing lines of pups differentiated from the randomly bred control line by the 3rd generation (Brunelli et al. 1997). At the 20th generation (Fig. 1), High line pups vocalized at more than 20 times the rate of Low line animals (Brunelli et al. 2010).

The selection process also altered physiological functions and other behaviors across the life span. Neonatal alterations included differences in birth weight, monoamine function, and urination/defecation. Juvenile changes were seen in autonomic regulation of heart rate and play behavior (Hofer et al. 2001; Brunelli 2005; Brunelli et al. 2010).

As adults, High line animals were characterized as having higher anxiety with sympathetic over‐activity (Brunelli 2005; Brunelli et al. 2010). Conversely, Low line animals were characterized as low anxiety, with parasympathetic underactivity and increased aggression. Low line mothers exhibited more licking and grooming. Following findings from other selective breeding programs (Koolhaas et al. 1999; Overstreet 2002; Steimer and Driscoll 2003; Burghardt et al. 2011), we have interpreted these changes as evidence for a “passive” coping style in the Highs and an “active” coping style in the Lows (Brunelli et al. 2002).

Developmental differences in ear canal opening (earlier for the High line) (Hofer et al. 2001; Brunelli 2005) led to questions about whether call rate might also be linked to other, nonmorphological acoustic traits. To determine whether there are any acoustic differences in the pups' isolation‐induced USV besides the breeding parameter, call rate (Fig. 1), we examined vocalizations for (1) duration; (2) amplitude; and (3) frequency bandwidth in High, Low, and Random lines after 36–39 generations. When breeding for a higher rate, we might expect animals to conserve energy expenditure by reducing other parameters like call duration and amplitude. Similarly, when breeding for a lower rate, we might expect animals to increase other parameters. Alternatively, the acoustic characteristics might be linked such that breeding for higher rate would result in increases in energy in some or all acoustic parameters, and breeding for lower rate would result in decreases. Or, a combination of these two possibilities might reflect an alternate mechanism.

## Materials and Methods

### Subjects

The experimental subjects were three lines of rat pups bred in our vivarium from the N:NIH strain, which is an outbred mix of eight commonly used laboratory rat strains. Pups were tested for vocalization rate during isolation at postnatal day 10 (PND10) and then selectively bred in adulthood. The criterion for selection was the number of USVs emitted during 2 min of isolation at PND10. One line was bred for a high rate of isolation‐induced vocalization (High). A second line was bred for a low rate (Low). The control line of pups was chosen randomly, independent of the rate of isolation‐induced vocalization (Random). Breeding was done between litters, not within (Brunelli et al. 1997; Brunelli and Hofer 2001). The rate of isolation‐induced USV by the selectively bred lines differentiated rapidly: The Low line was significantly different from Randoms in the 1st selected generation and the High line by the 3rd (S1 and S3, Fig. 1). Since that time, the separation between the lines increased and then maintained.

In this study, we tested litters from generations 36 to 39. There were 34 High pups from 11 litters; 15 Random pups from seven litters; and 27 Low pups from nine litters. Each litter supplied from 1 to 4 pups. All pups were PND10 ± 1 on the day of testing. The litters were housed with hardwood chip bedding in polycarbonate terraria (40 × 20 × 24 cm). Food and water was available ad libitum. Colony room temperature (~21°C) and humidity (~40%) were regulated. The light/dark cycle was 12/12 h with lights on at 7 am. The day of birth was counted as PND0. Large litters were culled to 10 pups on PND1. All litters had at least 8 pups. Except for normal husbandry, litters were left undisturbed following culling until the day of testing.

### Procedure

On the day of testing, the litter was taken in its home cage to the test room and placed on a thermoregulated heating pad (36.5°C). After 15–30 min of habituation, each pup in its turn was picked up from the home cage, had its axillary temperature taken, and placed alone in a novel container under room temperature conditions (~22.3°C). The observations began immediately after the animal was placed in the novel container.

Each experimental pup was observed in the novel container during an isolation of 2 min. The test containers were empty polycarbonate rectangular boxes (18 × 21 × 20 cm) with the floors marked to divide the area into eight equal squares. Pups had no previous experience with the test containers. Following each test, each pup's axillary temperature and weight were taken before being returned to its home cage. After the last pup was tested, the litter was returned to its dam in the colony room.

Behaviors noted included the number of floor squares entered (a measure of locomotion), rises (in which the head must be raised above shoulder level and at least one paw off the floor), turns‐in‐place (360° rotation without leaving a rectangle), self‐grooming, and defecation or urination (D/U) (Shair et al. 2005).

Ultrasonic vocalizations were transduced into the audible range using a bat detector (Pettersson Elektronik D 240×, Uppsala, Sweden) with its microphone suspended approximately 10 cm above the test container floor. Particular attention was paid to the distance of the pup from the microphone and its position toward the microphone to have comparable results. The detector was used in the heterodyne mode, tuned to 40 kHz. The experimenter wearing earphones counted USV pulses by pressing the button of a silent electronic counter. Number of pulses was measured as the number of distinct vocalizations in a two‐min period. Periodic inter‐rater reliability tests were performed in the laboratory to ensure that counting of USV is more than 90% reliable, as previously reported (Hofer and Shair 1978). A second experimenter recorded all observations in a time‐based format. Ambient temperature was monitored throughout the test with an air sensor positioned seven cm above the cage floor.

The signal from the bat detector was also collected with a sampling frequency of 100 kHz onto a computer for analysis by the BatSound Pro Program, version 3.3 (Pettersson Electronik AB, Uppsala, Sweden). BatSound Pro provided a spectrogram of the sounds for the test time period from which duration, amplitude, and bandwidth were calculated. In the present report, data from two‐min observation were run through high and low pass filters (30 and 55 kHz, respectively). Duration, amplitude and bandwidth were determined by identifying, marking, and measuring pulses in the spectrogram. Duration was measured as the length of time from the beginning to the end of each individual pulse. Amplitude measurements were relative, for purposes of comparing Lines and not for providing calibrated measurements of vocalization amplitudes. The minimum/maximum frequencies (bandwidth) were calculated as the highest and lowest points on the frequency spectrum for each individual pulse.

### Statistical analyses

Analyses were by ANOVA with Bonferroni post hoc tests (Systat Software Inc, Richmond, CA). Due to the issue of litter effects in developmental studies (Holson and Pearce 1992; Smotherman and Robinson 1994), ANOVAs used litter means as the unit of analysis. As much as possible, equal numbers of male and female pups were included in each condition. Possible effects of gender on USV were tested in all experiments by the inclusion of sex as a factor in preliminary ANOVAs. In no case did sex of the pup influence the rate of USV significantly, which replicates previous work on pups of this age (Brunelli et al. 1997; Shair et al. 2005), but contrasts to the report by Bowers et al. tested at a younger age (PND4) and using a different strain of rats (Bowers et al. 2013). Therefore, data from male and female pups were combined in the litter means. Pearson product‐moment correlations were used on individuals within each line to investigate relationships among the vocal parameters, as well as possible influences between the behavioral measures and USV rate.

## Results

**Figure 2:**
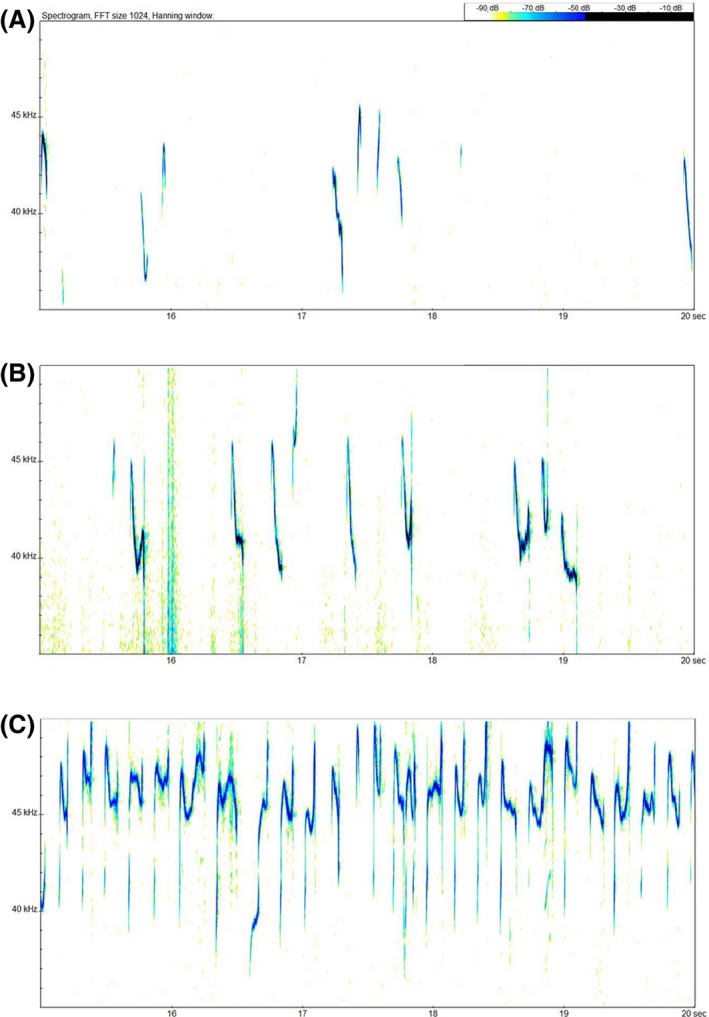
Examples of 5‐sec spectrograms of infant rat ultrasonic vocalizations during isolation for A, Low line, B Random line and C, High line. The High line makes more, louder, longer, and more broadband calls than the Low line (see Fig. 3 for analyses). Amplitude is indicated by the color of the spectrogram. Frequency bandwidth is measured from the maximum to minimum frequency in each vocalization. Samples were taken from the 15‐ to 20‐sec period after 10‐day‐old rat pups were isolated in a novel cage.

**Figure 3:**
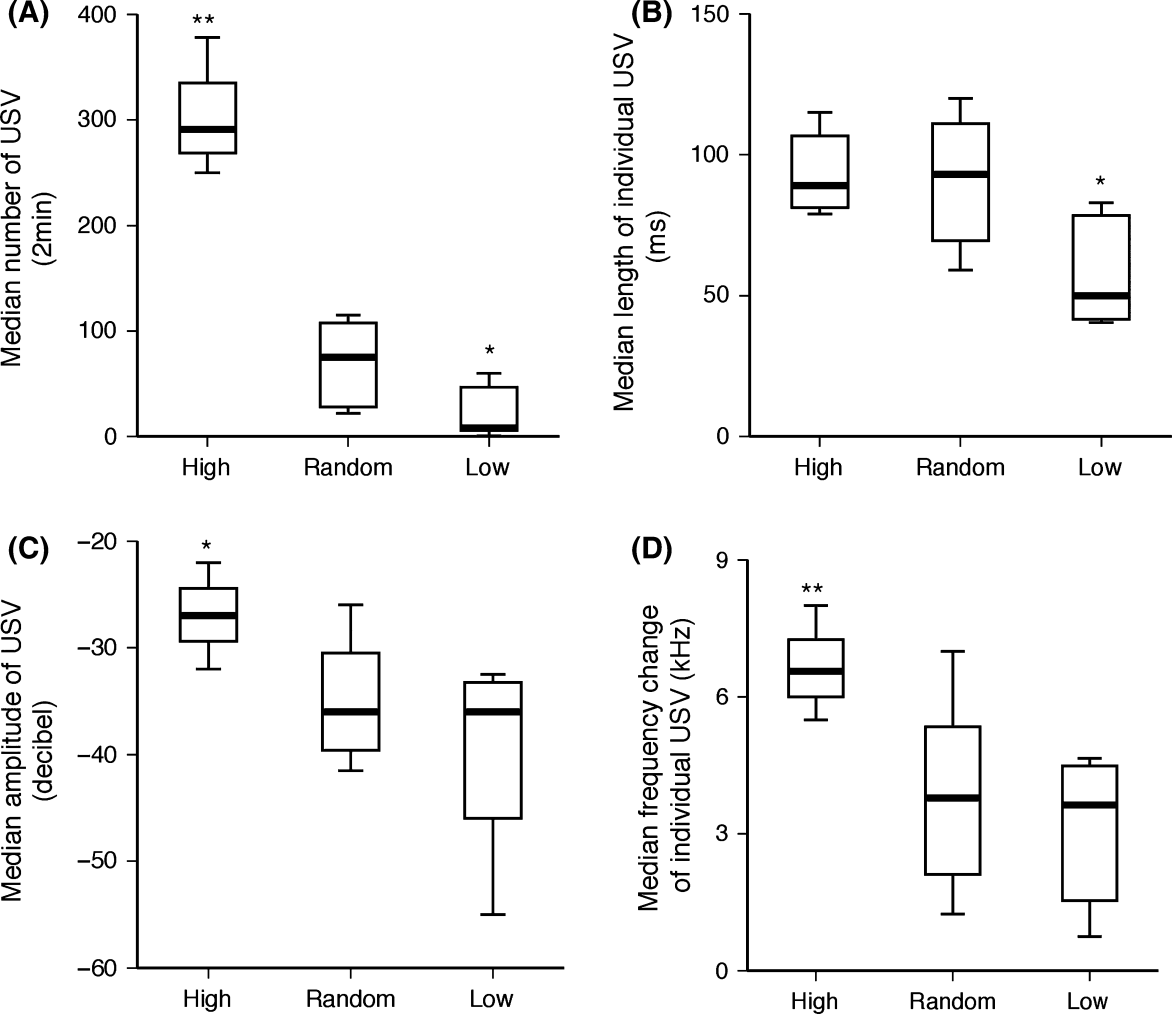
Box‐and‐whisker plot comparing acoustic parameters of calls for the two selectively bred lines and their random controls. Parameters are A, call rate; B, duration; C, amplitude; and D, frequency bandwidth of the ultrasonic vocalizations (USV). One‐way ANOVAs on litter means indicated significant effects of line in all four parameters: A, call rate *F*(2, 24) = 80.9, *P* < 0.001; B, duration *F*(2, 24) = 5.3, *P* < 0.05; C, amplitude *F*(2, 24) = 13.4, *P* < 0.001; D, call frequency bandwidth *F*(2, 24) = 18.2, *P* < 0.001. Bonferroni post hoc tests demonstrated that High and Low line groups differed significantly on all four parameters (*P* < 0.001). Cases in which one of the selectively bred lines differed from the random controls are indicated by a star (*P* < 0.05) or double stars (*P* < 0.001).

Selection for isolation‐induced rat pup USV rate coselected other vocal parameters. In our sample, the High line animals called at extremely high rates in isolation; the Low line animals produced very few USV; and the Random line was between the other two (Figs. 2, 3A). Line differences were also found for the other acoustic parameters measured. One‐way ANOVAs on litter means indicated significant effects of line in all four parameters: call rate (*F*(2, 24) = 80.9, *P *<* *0.001) (Fig. 3A); duration (*F*(2, 24) = 5.3, *P *<* *0.05) (Fig. 3B); relative amplitude (*F*(2, 24) = 13.4, *P *<* *0.001) (Fig. 3C); and call frequency bandwidth (*F*(2, 24) = 18.2, *P *<* *0.001) (Fig. 3D). Bonferroni post hoc tests demonstrated that High and Low line groups differed significantly on all four parameters (*P* < 0.001). The High line makes more, louder, longer, and more broadband calls than the Low line.

Contrary to an energy conservation hypothesis, High line animals were significantly higher than Low line on all three acoustic parameters measured. Compared to the Random line, High line calls increased significantly in amplitude (Fig. 3C) and frequency bandwidth (Fig. 3D). Low line pups emitted significantly shorter USV than Randoms (Fig. 3B). These results are consistent with a coselection hypothesis. The increased bandwidth of the calls in the High line, which gained higher frequencies, is particularly of interest because it has been hypothesized that frequency sweeps contain encoded information for the dam (Brudzynski 2005).

To create changes in USV frequency bandwidth like those in Fig. 3D, it is possible to gain or lose frequencies at both upper and lower extremes. The increased bandwidth in the High line was due to an increase at the upper end compared to the Random pups (mean maximum frequency High 44.52 ± 0.40 kHz, Random 41.56 ± 0.76 kHz; *F*(2, 24) = 4.69, *P *<* *0.05). Maximum frequency of Low pups was not altered significantly by selection (43.492 ± 0.86 kHz). Conversely, compared to the Random line, Low animals lost bandwidth at the bottom (mean minimum frequency Low 40.40 ± 0.88 kHz, Random 37.93 ± 0.53 kHz; *F*(2, 24) = 4.46, *P *<* *0.05). High line animals did not lose the lower frequency range (38.23 ± 0.40 kHz).

Correlations were used to look for relationships among the vocal parameters within each line. There are strong positive correlations in the Random line pups, but breeding for rate reduced correlations in the High line, and correlations were lost entirely in the Low line (Table 1). For example, the correlations of amplitude with number, duration, and bandwidth in the Random lines are all statistically significant, ranging from 80% to 97%. In the High line, the range drops to 39% to 72% with only two reaching significance. None of these correlations are significant in the Lows (37% to 51%). This pattern supports that selection for rate coselected the other parameters, as opposed to having had direct selection effects on them.

**Table 1:** Correlations among vocal parameters for each line using litter means as the unit of analysis

	Number	Duration	Amplitude	Bandwidth
High (*N* = 11)				
Number	–	–	–	–
Duration	0.05	–	–	–
Amplitude	0.39	0.71*	–	–
Bandwidth	0.65*	0.59^t^	0.72*	–
Random (*N* = 7)				
Number	–	–	–	–
Duration	0.44	–	–	–
Amplitude	0.83**	0.80*	–	–
Bandwidth	0.87**	0.68^t^	0.97**	–
Low (N = 9)				
Number	–	–	–	–
Duration	−0.04	–	–	–
Amplitude	0.51	0.37	–	–
Bandwidth	0.29	0.18	0.50	–

Asterisks indicate significant correlation at: ^t^ *P* < 0.1; *P < 0.05; **P < 0.01.

Correlations to USV rate were performed for all weight and temperature measures, as well as all observed behaviors in the isolation cage. None of these correlations reached significance for any of the three lines. The lines did differ on some behavioral measures, but all such differences were due to the selectively bred lines differing from the Random controls (Table 2). High and Low line groups did not differ significantly on any measure. Within each line, no behavioral measures correlated with USV rate either, which supports the lack of influence of behavior on USV rate.

**Table 2:** Behavioral data summary for each line. Values for each line are means (and SEMs) using litter means as the unit of analysis. Superscript symbols in the Bonferroni results indicate significant differences

Line	Wt (g)	Temperature measures		Observation measures		SqX	Rise	SG	TIS	D/U
		Preax (°C)	Postax (°C)	Preamb (°C)	Postamb (°C)					
High	19.6	35.2	33.8	21.3	21.5	1.1	0.1	0.2	0.5	0.1
SEM	0.83	0.35	0.16	0.22	0.25	0.29	0.05	0.08	0.14	0.07
Rand	22.1	36.1	33.6	21.3	21.3	3.4	0.6	0.9	0.64	0.4
SEM	0.9	0.13	0.19	0.11	0.31	1.6	0.29	0.36	0.34	0.16
Low	18.8	35.5	34.0	21.3	21.5	1.1	0.1	0.2	0.3	0.0
SEM	0.99	0.19	0.26	0.22	0.28	0.36	0.08	0.11	0.14	0.00
*F*	3.06	2.77	0.88	0.04	0.10	2.64	4.20	4.54	0.55	5.43
*P*	0.066	0.083	ns	ns	ns	0.092	0.027	0.021	ns	0.011
Bonf results							R > H*	R > H*		R > H^t^
							R > L^t^	R > L^t^		R > L**

High and Low lines do not differ significantly on any measure. Wt, body weight; g, grams; Preax, axillary body temperature immediately before testing; Postax, axillary body temperature immediately after testing; Preamb, ambient air temperature in the test chamber at start of test; Postamb, ambient air temperature in the test chamber at end of test; SqX, number of squares entered; Rise, number of rises; SG, number of episodes of self‐grooming; TIS, turns in square; DU, number of acts of elimination (See Methods for more description); R, Random line; H, High line; L, Low line; SEM, standard error of the mean; Bonf, Bonferroni post hoc test; ^t^ *P* < 0.10; *P < 0.05; **P < 0.01.

## Discussion

The High line pups have the potential to be better communicators, by transmitting more information per time unit and potentially farther. Every acoustic parameter measured changed in a direction favoring enhanced communication for the High line in comparison with the Low line. Higher call rates, longer call durations, and broader frequency ranges enable more information to be encoded and transmitted in the same amount of time. Louder calls, and possibly the other parameters, improve propagation and mitigate transmission loss. Conversely, Low line pups have characteristics that decrease communication potential. This difference in communication potential can also be examined in terms of energy expenditure, where the High line pups invest more energy in communication than the Low line pups. More calls, of longer duration, higher amplitude, and broader frequency range require more energy investment to produce. In the case of isolation‐induced calling, effective communication to the dam is necessary for survival (e.g., D'Amato et al. 2005; Ehret 2005; Hahn and Lavooy 2005; Thornton et al. 2005). Changes in one or more of these acoustic parameters could enhance or compromise effective communication.

There may be an adaptive reason to reduce the characteristics of vocalization in concert, as well as to increase them. Isolated pups can be heard by animals other than the mother, in particular by predators. Pups dramatically reduce USV rate when the presence of a predator is signaled. They also demonstrate other indicators of a fear response including immobility, urination, and increases in analgesic threshold and ACTH production (Takahashi 1992, 1994; Wiedenmayer and Barr 1998). The vocalization rate of randomly bred pups may be a compromise between these two pressures. Variations in predation levels may be one factor to favor a modular organization in acoustic traits that can respond efficiently to predation pressure (Wagner et al. 2007).

The acoustic parameters tested are specifically aspects of communication during a stressful situation, that is, isolation from the mother, and in addition to being coselected with each other, also appear to be closely tied to an underlying suite of emotion‐regulating traits that are accessible across the life span. A similar association of vocalization and emotional characteristics has been found in rat lines selected as adults for their call rate in response to “tickling” (Burgdorf et al. 2008, 2009; Harmon et al. 2008): the High line adult animals vocalize more to gentle tactile stimulation, a positive stimulus, as well as vocalizing less in isolation as pups; the opposite is true for their Low line. These authors characterize their High line as “stress resilient” and the Low line as “stress prone” phenotypes. This matches the present lines characterized as “low anxiety” or “active” coping (Low line) and “high anxiety” or “passive” coping (High line) (Brunelli et al. 2010). It appears that high vocal response in infants to a negative stimulus (isolation) corresponds with being high anxiety or stress prone, whereas high vocal response in adults to a positive stimulus (tickling) corresponds with being low anxiety or stress resilient.

In any selective breeding study from which a genetic or epigenetic mechanism is inferred, it is important to demonstrate that new selections for the same variables produce replicate results. We did not have the opportunity to perform a second experimental selection before the colony was discontinued. To rule out definitively that random chance produced the results in this report, such a replication is required. The separate selection requirements for the High and Low lines, however, are a type of replication. The fact that selection for high and low call rates produced changes of other acoustic variables in opposite directions strongly supports the coselection hypothesis.

It is possible that selection for rate could directly select for other acoustic parameters independent of functional linkage, if the parameters were highly correlated with rate in the progenitor line and thus served as additional selection parameters. In fact, in the Random line animals, highly significant correlations with rate were found for amplitude and bandwidth (Table 1). However, the changes seen in duration, amplitude, and bandwidth that accompany the changes in rate are very unlikely to be due to direct selection, because the correlations with rate were reduced or not significant in both High and Low lines. Breeding for rate broke the correlations, even though the parameters for the groups changed in concert with rate. Further work with larger numbers of animals is necessary to investigate whether there are any direct selection effects.

The coselective process established in this laboratory could also apply in wild populations. Animals must be prepared to survive under different conditions. Coselection of related parameters may provide one way to respond to environmental changes (Wagner et al. 2007). In a noisy environment, the more frequent, louder, longer, broader frequency calls of the High line pups would facilitate communication with the mother over those of the Low or Random line pups. As adults, the animals would presumably be faced by similar environmental noise pressures. Under such conditions, a higher level of anxiety (up to a point) may be beneficial as it ensures increased vigilance and rapid responsiveness. Taking together the critical role of dam–pup communication for survival, coselection of acoustic traits of vocalizations, and the stress responsivity patterns, selective pressures from increasingly noisy environments could have compounding, extensive effects on natural populations.

## Conflict of Interest

None declared.
